# A Novel Cytotoxic Conjugate Derived from the Natural Product Podophyllotoxin as a Direct-Target Protein Dual Inhibitor

**DOI:** 10.3390/molecules25184258

**Published:** 2020-09-17

**Authors:** Ángela-Patricia Hernández, Paula Díez, Pablo A. García, Martín Pérez-Andrés, Pablo Ortega, Pablo G. Jambrina, David Díez, María Ángeles Castro, Manuel Fuentes

**Affiliations:** 1 Departamento de Ciencias Farmacéuticas, Área de Química Farmacéutica, Facultad de Farmacia, CIETUS/IBSAL, Universidad de Salamanca, Campus Miguel de Unamuno, 37007 Salamanca, Spain; angytahg@usal.es (Á.-P.H.); pabloagg@usal.es (P.A.G.); 2 Department of Medicine and General Cytometry Service-Nucleus, CIBERONC CB16/12/00400, Cancer Research Centre (IBMCC/CSIC/USAL/IBSAL), 37007 Salamanca, Spain; pauladg@usal.es (P.D.); mmar@usal.es (M.P.-A.); 3 Proteomics Unit, Cancer Research Centre (IBMCC/CSIC/USAL/IBSAL), 37007 Salamanca, Spain; 4 Departamento de Química Física, Facultad de Ciencias Químicas, Universidad de, 37008 Salamanca, Spain; portega@usal.es (P.O.); pjambrina@usal.es (P.G.J.); 5 Departamento de Química Orgánica, Facultad de Ciencias Químicas, Universidad de, 37008 Salamanca, Spain; ddm@usal.es

**Keywords:** podophyllotoxin, etoposide, protein inhibition, tubulin polymerization, anti-topoisomerase-II, hybridization, conjugation, cytotoxicity, flow cytometry, molecular docking

## Abstract

Natural products are the ideal basis for the design of novel efficient molecular entities. Podophyllotoxin, a naturally occurring cyclolignan, is an example of natural product which displays a high versatility from a biological activity point of view. Based on its unique chemical structure, different derivatives have been synthesized presenting the original antitumoral properties associated with the compound, i.e., the tubulin polymerization inhibition and arising anti-topoisomerase II activity from structural modifications on the cyclolignan skeleton. In this report, we present a novel conjugate or hybrid which chemically combines both biological activities in one single molecule. Chemical design has been planned based in our lead compound, podophyllic aldehyde, as an inhibitor of tubulin polymerization, and in etoposide, an approved antitumoral drug targeting topoisomerase II. The cytotoxicity and selectivity of the novel synthetized hybrid has been evaluated in several cell lines of different solid tumors. In addition, these dual functional effects of the novel compound have been also evaluated by molecular docking approaches.

## 1. Introduction

**Figure 1:**
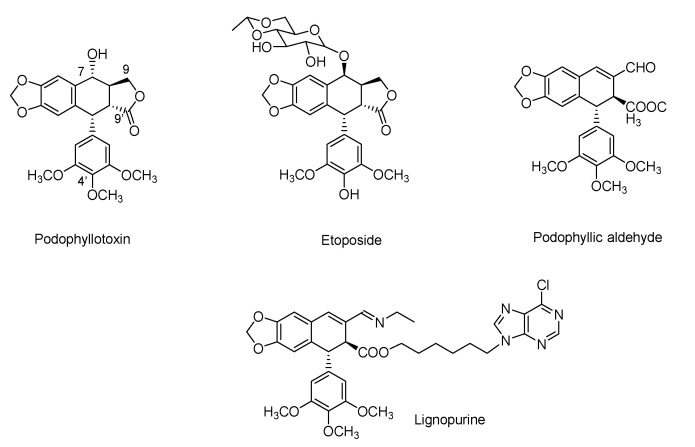
Chemical structures of natural product podophyllotoxin, clinically approved derivative etoposide, non-lactonic cyclolignan podophyllic aldehyde and lignopurine, a hybrid with a purine moiety.

In recent decades, the potential bioactivity of natural products has become well-known and well-characterized. Hence, the singularity of these compounds on the mechanisms of actions is based on three main characteristics: chemical skeletons, chiral structures and molecular complexity [1,2]. Moreover, these properties have turned natural compounds to potential therapeutic candidates in different pathologies, where their application in the treatment of cancer stands out [3,4]. The interaction of natural products with proteins leads to changes in or modifications to the proteins’ function, which can be exploited for therapeutic purposes. However, it is challenging to design a strategy for these molecules that could lead to new compounds with higher efficacy on the targeted protein than the parent compound. Podophyllotoxin (Figure 1), a naturally occurring compound, is one of this huge number of compounds that presents a specific interaction site with proteins. In fact, this cyclolignan, isolated from genus *Podophyllum*, presents a tubulin interaction at the colchicine binding site that inhibits its polymerization, destabilizing and preventing the formation of microtubules required for cell division [5]. The effect of this interaction turned podophyllotoxin into a recognized cell death inducer, an approved antiviral drug due to its cytotoxic activity [6].

As occurs with most natural products, structure–activity relationship (SAR) studies on the cyclolignan skeleton have been previously reported. From these reports, etoposide emerged as a new drug derived from podophyllotoxin that presented a particularity in its activity. The structural changes performed on the cyclolignan skeleton to obtain etoposide modified its mechanism of action, with it becoming an inhibitor of DNA-topoisomerase II (Topo-II) [7]. The chemical modifications made to podophyllotoxin, responsible for that change, include a demethylation in the trimethoxyphenyl pendant ring and the introduction of a bulky substituent with inverted configuration at C-7 position (Figure 1). The optimal biological properties of this compound caused etoposide to be approved as an anti-tumoral drug for several human tumors [7].

However, it is not only these structural changes that attribute novel properties to podophyllotoxin derivatives. The chemical skeleton of this cyclolignan has been widely modified, including different functional groups and heterocycles [8,9]. From those studies, the γ-lactone ring of the compound, which was considered an essential part for the cytotoxic activity, was also modified, leading to compounds with interesting profiles of potency and selectivity against several cancer cell lines. This is the case of podophyllic aldehyde (Figure 1), a non-lactonic derivative that showed improved cytotoxic results, reaching potency in the nanomolar range similar to that of the parent compound but with better selectivity than podophyllotoxin towards certain tumor cell lines [10,11]. It shares the same mechanism of action as podophyllotoxin. Recent chemical approaches performed in our group allowed us to prepare new conjugates or hybrids with other types of natural products (i.e., purines and terpenylquinones) (Figure 1), modulating the selectivity of the podophyllotoxin moiety [12,13]. Molecular conjugation or hybridization, as a tool in drug discovery, allows known compounds to improve their pharmacokinetic and pharmacological properties in an easy way. The combination of two skeletons from different origins is also common in natural products where structures with carbons from different secondary metabolic pathways can be found. The conjugation of structures yields compounds that may synergically improve the affinity and efficacy of original drugs [14,15,16,17].

Exploring the properties of the inversion of the configuration at position C-7 of podophyllotoxin structure, a huge structure–activity relationship has been carried out, taking hybridization as a tool and combining the cyclolignan skeleton with other compounds with different pharmacological activities, and obtaining interesting results on the bioactivity [18,19,20,21,22,23,24,25,26,27].

Drug discovery must be firstly supported by rational design, but also by a complete subsequent evaluation of the activity. In this context, cytomic and proteomic techniques are essential to confirm the success of the design [28,29,30] and, especially, flow cytometry has given very satisfactory results in terms of drug evaluation [31,32], including podophyllotoxin derivatives [13].

According to the previous experience of the group with cyclolignans and taking into account that protein–drug interaction requires a difficult optimization, we have planned a new family of hybrids, named biscyclolignans, that combine, in a single molecule, both structure requirements to directly target tubulin and Topo-II, taking advantage of the duality of the skeleton of podophyllotoxin.

**Figure 2:**
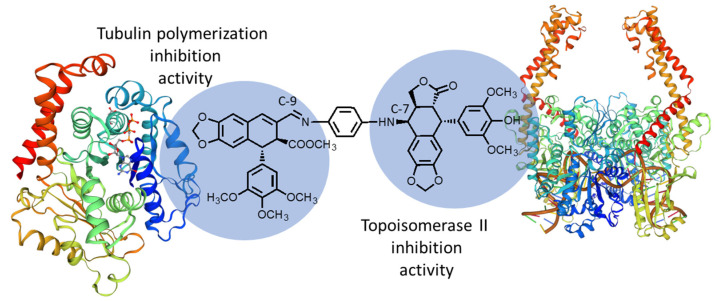
Schematic representation of 7β,9-biscyclolignan, formed by two cyclolignan fragments connected through a diamine spacer.

Therefore, we designed the first conjugate of this family, named 7β,9-biscyclolignan, formed by two cyclolignan fragments, one derived from 4′-*O*-demethylepipodophyllotoxin as Topo-II inhibitor and the other from podophyllic aldehyde as tubulin polymerization inhibitor (Figure 2). Both fragments can be connected through the C-7 and C-9 positions, respectively, through different linkers. In this case, we chose diamine type spacers, taking advantage of previous experience in the formation of imines in the aldehyde function [11]. Based on our previous results with hybrids of podophyllotoxin [13], in this paper, we report the first example of these new hybrids, in which the linker between both fragments is an aromatic diamine.

## 2. Results

### 2.1. Chemistry

In our previous synthesized compounds, the cytotoxic hybrids derived from podophyllic aldehyde were synthesized with the linkers attached to the cyclolignan at C-9′ position through ester-type bonds (Figure 1) [12,13]. In those previous works, an improvement in the potency and selectivity was observed for several imino derivatives of podophyllic aldehyde, which also act as inhibitors of the tubulin polymerization. Additionally, and as mentioned in the introduction, there are drugs such as etoposide and its derivatives that have very important cytotoxic properties acting by a different mechanism of action than podophyllotoxin and podophyllic aldehyde, promoted by the structural modifications in the cyclolignan structure [7]. Those facts prompted us to combine, in the same structure, both mechanisms of action. Thus, we describe here the design, synthesis and biological evaluation of the first conjugate of the new family of biscyclolignans.

Podophyllotoxin, the natural product, is the starting material of both cyclolignan fragments present in the final conjugate. The first step in the synthesis is the isolation of podophyllotoxin from commercial resin of *Podophyllum emodi* (actually *Sinopodophyllum hexandrum* (Royle) T.S.Ying, family *Berberidaceae*) by a previous procedure optimized by us [12]. Once the podophyllotoxin, **1**, was purified (40%), both cyclolignan fragments could be obtained.

On one side, for the podophyllic aldehyde rest, the γ-lactone ring of podophyllotoxin was opened in basic conditions and the carboxylic acid was esterified with a methyl group. Oxidation under Swern conditions led to the podophyllic aldehyde, **2** [33,34].

**Scheme 1:**
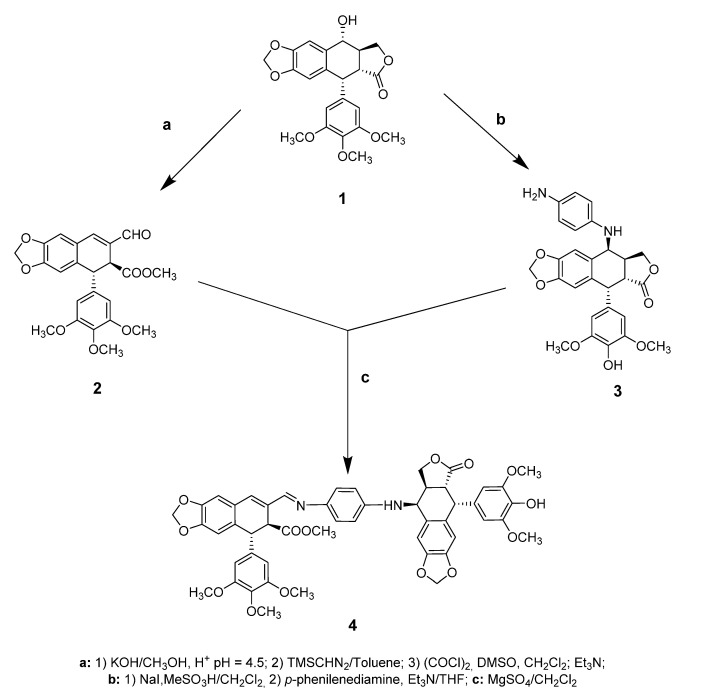
Synthesis of the dual protein inhibitor 7β,9-biscyclolignan derived from podophyllotoxin.

On the other hand, *O*-demethylation at the C-4′ and epimerization of the C-7 position of podophyllotoxin, **1**, followed by replacement by an aromatic diamine-type linker, gave rise to the other fragment of the hybrid, **3**, in a one-pot reaction [35]. Finally, aldehyde **2** was condensed with the 7β-cyclolignan **3** through an imine-type bond, obtaining the final 7β,9-biscyclolignan **4** (Scheme 1).

### 2.2. Biological Assays

#### 2.2.1. Evaluation of Cellular Viability

Once the precursors, **2** and **3**, and the final hybrid **4,** were purified and characterized, we investigated the evaluation of the effect of these compounds in the cellular viability, in particular the inhibition of tubulin and Topo-II activity, which lead to cell death as a consequence of their activity. Thus, quantification of the cell viability is necessary to determine the potential activity of the 7β,9-biscyclolignan **4**.

**Figure 3:**
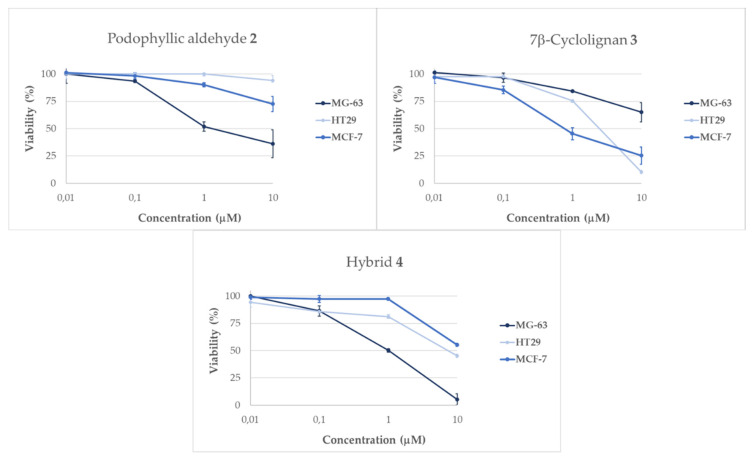
Results obtained from viability assays performed in tumor cells lines MG-63 (osteosarcoma), HT-29 (colorectal cancer) and MCF-7 (breast cancer) for the compounds **2**, **3** and **4** in different concentrations after 24 h of incubation.

For this study, the podophyllic aldehyde, **2**, and the anti-topoisomerase derivative **3** and the hybrid **4** were tested on three different human cell types: osteosarcoma (MG-63), colorectal (HT-29) and breast cancer (MCF-7) cell lines. The cytotoxicity against them was evaluated at different concentrations. Several observations about the behavior of the compounds can already be highlighted in this assay, as shown in Figure 3.

The evaluation of cytotoxicity across the three human tumoral cell lines displayed differences at 24 h incubation for all the tested compounds. Podophyllic aldehyde, **2**, was more potent in MG-63 cell line, while the MCF-7 cell line was slightly affected by this compound, and HT-29 was practically unaffected after 24 h. These results of our lead compound are in accordance with previous works and confirm the selectivity of this compound in comparison with the natural product podophyllotoxin, **1** [13]. Regarding the 7β-precursor **3**, there were two cell lines very sensitive to this compound, HT-29 and MCF-7, while the osteosarcoma cell line MG-63 presented a reduced affectation compared with the other two. These differences in behavior show that both precursors are well-differentiated in their activity, as expected from the structural–activity relationship of the cyclolignans [5]. Moreover, a different activity pattern can be observed for the final hybrid **4**. This compound showed remarkable activity in the osteosarcoma cell line, being more potent than its precursors in this cell line. With these initial results, we observed the efficiency of the hybridization in cyclolignans and the potential of the new conjugate **4**, which improved the activity of the precursors in the osteosarcoma cell line.

#### 2.2.2. Apoptosis Evaluation

**Figure 4:**
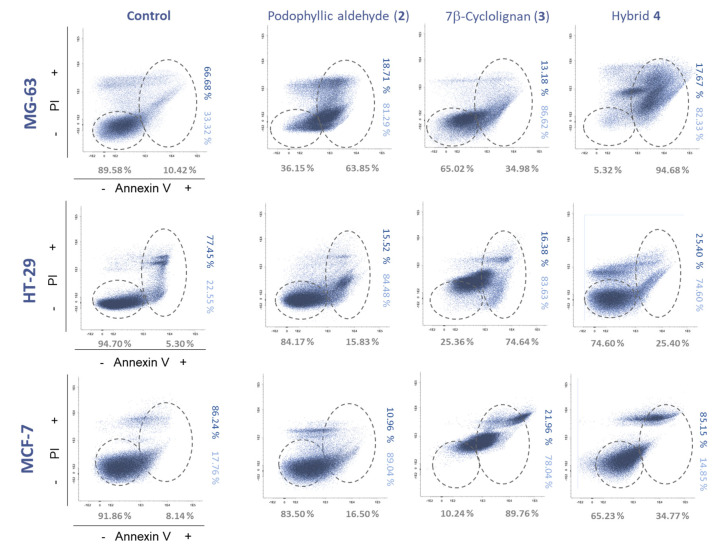
Apoptosis assays performed by flow cytometry with the compounds **2**–**4** in the cell lines MG-63 (osteosarcoma), HT-29 (colorectal cancer) and MCF-7 (breast cancer) at 24 h of incubation at 10 μM and control with vehicle (DMSO, <0.001%). PI, propidium iodide. % Annexin V−/+ (grey), annexin V+/PI− (light blue) and annexin V+/PI+ (dark blue).

Apoptosis triggering is an important approach for determining the pattern of cell death mediated by the compounds. In this case, annexin V was used as a marker in flow cytometry for the determination of the cells in apoptosis stage, in combination with propidium iodide (PI), which detects late apoptosis and cell necrosis (Figure 4, Table S1). The analysis was performed in the three cell lines used in the viability analysis at 10 μM concentration.

These analyses gave interesting results for the tested compounds. The first compound, podophyllic aldehyde, **2**, showed a modest apoptosis effect, which is in accordance with our previously reported studies where we observed that the initiation of inhibition of tubulin polymerization in this compound and podophyllotoxin, **1**, did not show total cell death until longer incubation times [13].

The most affected cell line by this precursor was MG-63 with a 63.85% of annexin^+^ cells. The other cell lines tested only increased apoptotic cells by approximately 10% compared to the control, although dot-plots with different cell distributions were observed on the HT-29 and MCF-7 cell lines.

Interesting results were obtained for the 7β-cyclolignan precursor **3**, since a strong variation compared to the control was observed, as shown in Figure 4. These results could suggest a different mechanism of action of this precursor in comparison with the podophyllic aldehyde, **2**. Finally, the most outstanding results were obtained by the final conjugate **4**. MCF-7 cell line, whose viability was not affected by compound **4**, showed a cell distribution related to that of compound **3** by flow cytometry. This fact indicates that activity related to topoisomerase inhibition is present in this conjugate. Although this result is important, the values obtained for the MG-63 cell line draw much more attention where almost all the cells are in apoptosis (94.68% annexin^+^ cells), with a behavior closer to that of aldehyde **2**. This particular value shows that the combination of both cyclolignan moieties in the same structure triggers an intense cellular response that could be mediated by the addition of the two mechanisms of action involved in cyclolignan structures.

#### 2.2.3. Cell Cycle Evaluation

Since the inhibition of tubulin polymerization and the inhibition of Topo-II have a critical role in the cell cycle, we evaluated this to get more insights about the potency of the compounds tested.

**Figure 5:**
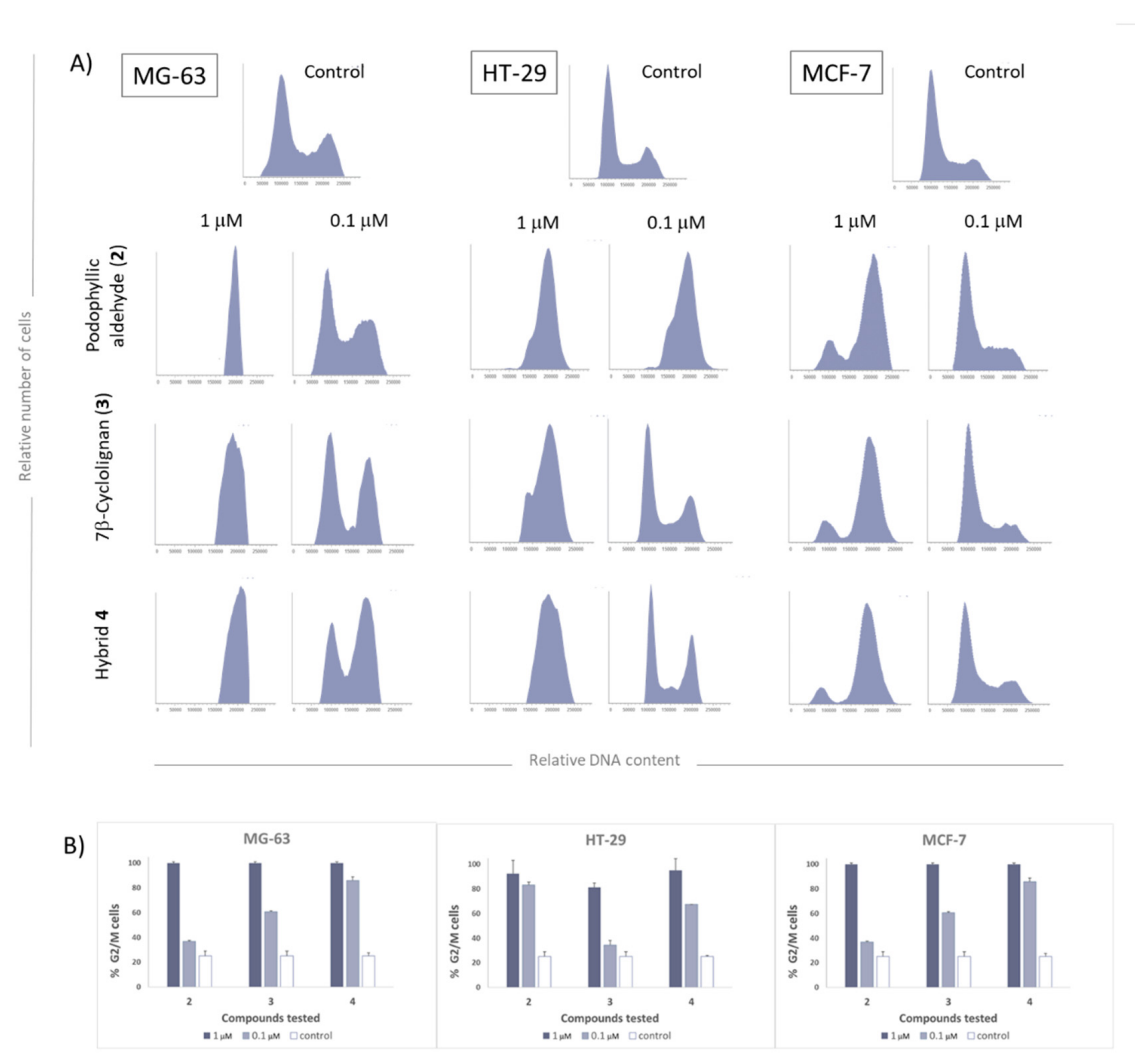
Cell cycle results of the compounds **2**–**4** in the cell lines MG-63 (osteosarcoma), HT-29 (colorectal cancer) and MCF-7 (breast cancer) at 24 h of incubation at 10 μM. (**A**) Flow cytometry diagrams (**B**) Cells (%) in G2/M phase dose depending.

Bearing in mind previously reported studies, the cell cycle arrest by podophyllotoxin and non-lactonic cyclolignans such as podophyllic aldehyde, is reached in the range of µM and sub-µM concentrations [11,12]. The results obtained in the cell cycle analysis are collected in Figure 5, where it is observed that the inhibition of both protein functionalities is characterized by a strong disruption of the cell cycle in the G2/M phase.

As can be observed in Figure 5, at µM range, all these tested compounds are biologically active in all the three studied human cell lines. In the case of MCF-7, it was less sensitive, while MG-63 was the most affected one. Neither the precursors nor the final compound affected the MCF-7 cell cycle at 0.1 μM; however, a partial arrest of the cell cycle was observed when the concentration was 10x increased.

HT-29 cell line was usually the most affected by other compounds synthetized by our group [12,13,31], like podophyllic aldehyde, **2**, and this tendency is confirmed in this study. The final conjugate also shows increased biological activity in this colorectal cancer cell line, which may be attributed to tubulin inhibition activity.

Finally, the MG-63 cell line seems to present a higher disruption of the cell cycle even under µM range. In this cell line, both precursors have shown activity below μM range and the final hybrid **4** was the most potent in arresting the cycle at the G2/M phase. These results suggest that the final conjugate **4** is able to act simultaneously in both targeted proteins, reaching our objective of merging both mechanisms of action in a single molecule.

### 2.3. Molecular Docking Studies

Several molecular dynamics (MD) and docking calculations have been carried out in order to obtain structural evidence that compound **4** can act as an inhibitor for the targeted proteins. As described in the methods section (Section 4.3), we set up the simulations based on the 1SA1 and 5GWK structures in which tubulin is co-crystallized in the presence of podophyllotoxin, and Topo-II is co-crystallized with etoposide in its active site. For each of the systems, four different simulations were performed that differ in the ligand that is included: podophyllotoxin, etoposide, podophyllic aldehyde, or hybrid **4**. After the MD simulation, interaction energies were calculated using the NAMD-Energy plugin, and binding free energies were recorded using Autodock-VINA [36].

#### 2.3.1 Tubulin Interaction

Podophyllotoxin has been described to occupy the colchicine binding site. This pocket is embedded in the β-subunit, close to the interface with α−tubulin and GTP binding site. It is thought that ligands that occupy the colchicine binding site inhibit tubulin dimerization via dimer destabilization, thus preventing the required contacts of the protomers from forming the microtubule [37,38].

In Table 1, the binding energies (ΔG) for the interaction of the four compounds in the colchicine binding site are shown. All the compounds interact strongly with the tubulin dimer, (−ΔG > 8.5 kcal/mol), in particular compound **4** for which ΔG = −12.5 kcal/mol. Interestingly, our results predict that the binding energy of **4** is even higher than that of podophyllotoxin, the reference compound.

**Figure 6:**
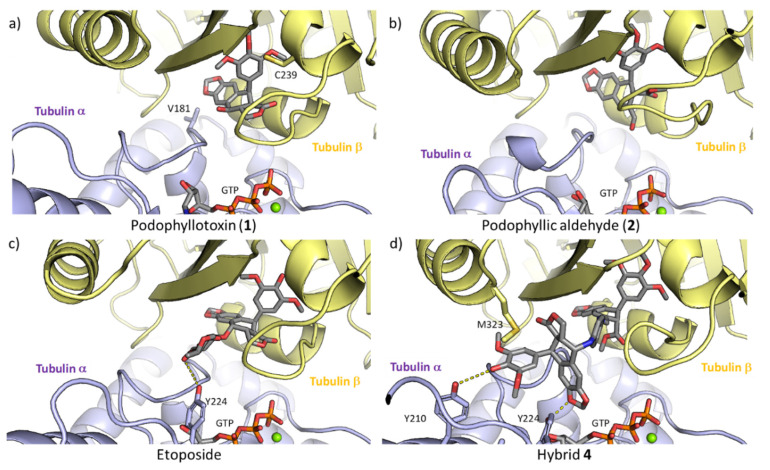
Docking complexes of compounds in the colchicine site of tubulin. (**a**) podophyllotoxin (**1**), (**b**) podophyllic aldehyde (**2**), (**c**) etoposide and (**d**) hybrid **4**. GTP, the inhibitors, and those residues that interact with the inhibitors via hydrogen bonds are shown atomistically. The two protomers tubulin α and β are shown in yellow and purple, respectively.

To explore the effect that the ligands have on impairing dimerization of tubulin, we calculated the interaction energies between α-tubulin and β-tubulin throughout the MD simulations. Our results showed that there was a significant decrease in the interaction energies when the new hybrid **4** occupies the colchicine site. These results suggest that the compound **4** is able to hinder the formation of the dimer and the subsequent polymerization of the tubulin to form microtubules better than the precursors. This is a consequence of the larger size of compound **4**, which fits in the dimerization interface, interacting with both α-tubulin and β-tubulin (Figure 6d). The interaction with tubulin, for the compounds considered, is depicted in Figure 6.

Podophyllotoxin (**1**) fits in the β-tubulin strand placing the trimethoxyphenyl residue in the inner part of the pocket while the four fused rings are located closer to the interface of the two protomers (Figure 6a). The same trend is followed by compound **2** (Figure 6b), as expected for the structural similarity. In the case of etoposide, the cyclolignan moiety is located in the same orientation as podophyllotoxin, but the disposition that etoposide adopts in the pocket does not interact with β-strand through the trimethoxyphenyl moiety in the same manner as the natural product. The glycosidic substituent at the C-7β position of etoposide enters the interface between the two subunits. In case of the new conjugate **4**, the interaction with the β-tubulin with its podophyllic aldehyde rest is maintained and the aromatic linker and the other cyclolignan moiety of the molecule are located in the dimerization interface, where it interacts with two tyrosine residues of α-tubulin (residues Y210 and Y224), thus justifying the decreased dimerization energy where **4** is bound to tubulin, and also its higher binding energy.

#### 2.3.2 DNA-Topoisomerase II Interaction

Additionally, DNA-topoisomerase αII isoform has been studied to further explore the complementary activity of the new conjugate **4**. According to previous studies [39], two etoposide molecules are able to interact with the DNA–protein complex so that they are located at both ends of the DNA breakdown. In this way, it inhibits the activity of the enzyme, preventing the re-ligation and maintaining the break of the DNA strands. The increase in the concentration of the Topo-DNA cleavage complexes leads to the death of cancer cells, which are not able to replicate their DNA material. Both binding sites are far enough in the crystal that the two ligands do not interact, and it seems fair to consider that binding of the first molecule should not affect the binding of the second ligand. In Table 2 we report the docking free energies after the MD simulation, and the interaction energies between the ligand and the DNA chains or the protein. The binding energies are high for all the compounds, due to the strong interaction between the ligand and the DNA chains in which they are intercalated. In particular, they are stronger for etoposide and hybrid **4** (ΔG < −12 kcal/mol).

**Figure 7:**
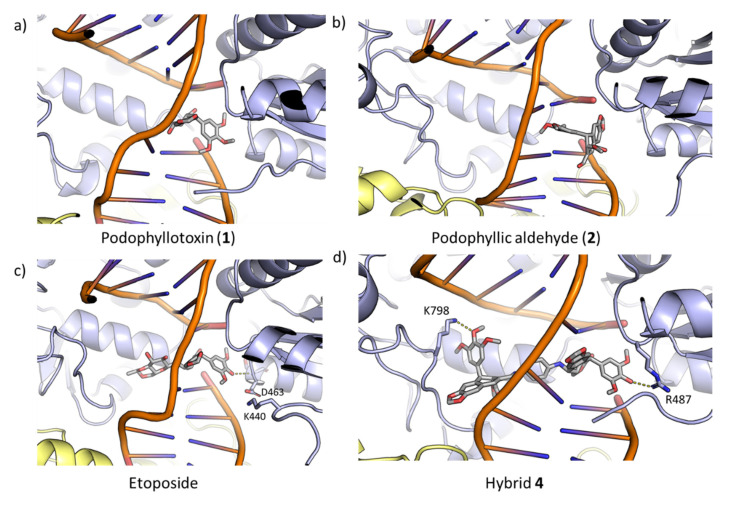
Docking complexes of compounds at end of the DNA breakdown. (**a**) podophyllotoxin (**1**), (**b**) podophyllic aldehyde (**2**), (**c**) etoposide and (**d**) hybrid **4**. The inhibitors, and those residues that interact with them via hydrogen bonds, are shown atomistically. The two subunits of topo-II are shown in yellow and purple, respectively.

As expected, podophyllotoxin and podophilic aldehyde have obtained lower energy values (ΔG) than etoposide, since the inhibition of Topo-II is not their pharmacological target; however, the energy found for the new hybrid **4** was highly similar to the one of etoposide. Likewise, for these compounds, **1** and **2**, lower values were obtained for the interaction with the DNA and, especially, with the protein. When the simulation of etoposide was carried out, a higher value for the interaction with the DNA was obtained. This result agreed with previously reported studies of this compound, where it was described that etoposide presents a high affinity to the nicked DNA strand [40]. The structure of the protein–DNA–ligand complexes after the MD simulations is shown in Figure 7.

Taking etoposide as the reference for Topo-II inhibition, it can be observed that the molecule is located in the middle of the DNA break. In addition, the dimethoxyphenyl ring interacts with the protein through a hydrogen bond between the main-chain of S462 and the hydroxyl at C-4 position. This interaction seems to be responsible for the interaction with the protein. As expected, podophyllotoxin and podophyllic aldehyde do not present an optimal disposition for interaction either with DNA or with protein, which justifies the lower values obtained in the calculations carried out (Table 2). In addition, it should be noted that in the case of podophyllic aldehyde, the trimethoxyphenyl ring is located between the two DNA strands.

Conjugate **4**, whose binding free energy is equivalent to that for etoposide, adopts an optimal disposition for interaction with DNA, remaining perfectly intercalated between the two strands resulting from the break. Regarding the interactions with the protein, it interacts with two basic residues of the protein (K798, and R487), thus leading to a stabilization of the complex. This fact justifies the considerable increase in the energy of the ligand–protein interaction that compound **4** presents with respect to the other compounds.

## 3. Discussions

A combination of two different podophyllotoxin derivatives in a single entity has originated a novel hybrid compound which shows, simultaneously, the dual mechanisms of action associated with cyclolignans. The chemical requirements for tubulin and Topo-II inhibition were successfully included in the same molecule. This novel hybrid combines the best modifications carried out in the non-lactonic podophyllotoxin derivatives [11] with the etoposide structural characteristics, such as a bulky substituent with inverted configuration in C-7 position and the *O*-demethylation in C-4′ [7]. The chemical design gave the desired compound and the highly analyzed activity yielded very consistent values regarding its cytotoxic activity. Comparing the precursors **2** and **3** integrated in the structure of the final hybrid **4**, the new conjugate presented the best values in viability, improving the precursors’ results on the osteosarcoma cell line. This is an important result that displays how the two structures may contribute to the final activity of the conjugate and shows the potential of the novel dual conjugate. The extensive flow cytometry analysis confirms the expectations of the viability results.

The evaluation of cell death effect reveals two different behaviors for these compounds. While tubulin inhibition is reflected by an increment in apoptotic cells (annexin^+^), the anti-topoisomerase activity is represented by an alteration in the cellular distribution in the cytometer graphic. Final compound **4** presented a total distortion in the distribution of cells in the dot plot according to the dual behavior implied in the conjugate, reaching the highest annexin^+^ value among the tested compounds. Furthermore, cell cycle inhibition was an important parameter to determine the activity of the compounds and the dual behavior of conjugate **4**, showing the highest potency in cell cycle arrest with a disruption mediated by the addition of two mechanisms: tubulin and topoisomerase inhibition. From the *in c* evaluation, the remarkable selectivity found in the tumor lines tested should be noted, where the best results were obtained in the osteosarcoma line compared to the other solid tumor lines tested. These results are interesting because osteosarcoma is particularly aggressive, presenting a high risk of metastasis [41]. Finally, docking studies have confirmed the potential of the new conjugate to interact directly with both proteins, showing the success of the design of the new conjugate and emphasizing that both mechanisms of action are present as deduced from the in vitro evaluation carried out. Further studies to increase the number of hybrids of this family (biscyclolignans) will be necessary for more in-depth SAR studies.

## 4. Materials and Methods

### 4.1. Chemistry

^1^H and ^13^C NMR experiments were recorded on Bruker Advance 400DRX (400 or 100 MHz respectively) spectrometers in CDCl_3_ using the residual solvent signal as reference. Chemical shift (δ) values are expressed in ppm, followed by multiplicity and coupling constants (*J*) in Hz.

Podophyllotoxin (**1**) was isolated from commercial resin of *P. emodi* following the protocol previous described by us [11].

Podophyllic aldehyde (**2**) was synthesized from podophyllotoxin as described in our previous works [35].

7β-Cyclolignan **3**: compound **1** (100 mg, 0.24 mmol) in dry CH_2_Cl_2_ (2.5 mL) stirring with NaI (160 mg, 1.08 mmol) for 15 min at room temperature (rt) and N_2_ atmosphere. MeSO_3_H (35 μL, 1.1 mmol) was dropped while the reaction was maintained in an ice bath. The mixture was stirred at rt for 5 h, then N_2_ was bubbled and the solvent was evaporated. The raw reaction was dissolved in dry THF (6 mL) and *p*-phenylenediamine (108 mg, 0.96 mmol) and Et_3_N (348 μL, 1.23 mmol) were added, stirring the mixture for 19 h at rt. The crude product was diluted with EtOAc and the solution was washed with saturated aqueous NaCl, dried over Na_2_SO_4_, filtered, and the solvent was removed under vacuum. The crude product was chromatographed on silica gel, eluting with 30% EtOAc/CH_2_Cl_2_ to yield compound **3** (24 mg, 20%).

7β,9-Biscyclolignan **4**: A mixture of aldehyde **2** (50 mg, 0.12 mmol), anhydrous MgSO_4_ (144 mg, 1.2 mmol) and amine **3** (58 mg, 0.12 mmol) was dissolved in CH_2_Cl_2_ (3 mL) and stirred at rt for 3 d. The reaction mixture was diluted with CH_2_Cl_2_, filtered and concentrated under vacuum. The crude product was chromatographed on silica gel, eluting with 20% EtOAc/CH_2_Cl_2_ to yield compound **4** (18 mg, 21%). See Table S4 and Figure S2.

### 4.2. Biological Methods

#### 4.2.1. Cell Lines and Culture Conditions

The human colorectal adenocarcinoma (HT29, ATCC HTB-38), the human breast cancer (MCF7, ATCC HTB-22) and the human osteosarcoma (MG-63, ATCC CRL-1427) cell lines were grown in adherent monolayer culture in Dulbecco’s Minimum Essential Medium (DMEM) medium for HT29 and MCF7 cells and RPMI medium for MG-63, and both were supplemented with 10% heat-inactivated fetal bovine serum (FBS), 1% L-glutamine and 1% penicillin/streptomycin in a humidified atmosphere (5% CO_2_, 37 °C). All media and supplements were purchased from Sigma (St. Louis, MO, USA).

#### 4.2.2. Flow Cytometry Assays

Cell viability assays and apoptosis detection by annexin V/propidium iodide assay.

To determine cell growth inhibition, early and late apoptosis, as well as cell death, the annexin V/propidium iodide assay (ImmunoStep, S.L., Salamanca, Spain) was applied. A total of 200,000 cells were seeded in 35-mm cell culture dishes (Falcon, Corning, NY, USA) and allowed to settle for 24 h. Then, serial dilutions of the chemical compounds (10, 1, 0.1 and 0.01 μM) were added to expose the cells for 24 h. After the incubation period, cells were detached by trypsinization and collected into flow cytometry tubes together with the supernatants. After three washing steps with PBS (1200 rpm, 3 min), 1X annexin-binding buffer was added to re-suspend the cells at a 1 × 10^6^ cells/mL concentration. Then, cells were systematically stained with 5 μL of the annexin-V fluorescein isothiocyanate (FITC) and 5 μL of the propidium iodide and incubated for 15 min at room temperature in the dark. After this time, 400 μL of 1X annexin-binding buffer was added. For data analysis of the annexin+/− and PI+/− ratios, the Infinicyt™ software (Cytognos SL, Salamanca, Spain) was used. By representing viability (% annexin− cells) on the vertical and concentrations of the compound on the horizontal one, curves of viability were obtained.

#### 4.2.3. Cell Cycle Analysis

To study the cell cycle of HT-29, MCF-7 and MG-63 cells, a solution containing ribonuclease (RNase), propidium iodide (PI) and detergent was added to the cells. As described for the apoptosis assay, cells were collected into flow cytometry tubes and washed three times with PBS (1200 rpm, 3 min). Then, 200 μL of the solution mentioned above was added to each tube and incubated for 10 min at room temperature in the dark.

In both flow cytometry assays, data acquisition was performed in a FACS Canto II flow cytometer (Becton Dickinson Biosciences, BD, San Jose, CA, USA) using the FACS Diva software (v6.1; BD). For data analysis, the Infinicyt™ software (Cytognos SL, Salamanca, Spain) was used.

### 4.3. Computational Methods

Our models for tubulin dimer and Topo-II were based on the PDB 1SA1 and 5GWK [37,38], respectively. In 1SA1, the podophyllotoxin was resolved at the colchicine site, close to the dimerization interface. 5GWK corresponds to the ternary complex between human Topo-II, the DNA strands and two molecules of etoposide. Homology modeling was carried out using SWISS-Model [42,43], using these two structures as a template. For each of the two proteins, we prepared four different simulations with either podophyllotoxin, etoposide, podophyllic aldehyde or **4** in the pockets occupied by podophyllotoxin and etoposide in 1SA1 and 5GWK, respectively. For the latter case, two ligand molecules were included in the simulation, replacing the two molecules of etoposide resolved in 5GWK.

Once the complexes were prepared, they were placed in the center of a cubic water box large enough to contain the protein and at least 10 Å of solvent on all sides. To mimic the intracellular conditions, K^+^ and Cl^-^ were added to the system to account for a 0.15 mol/L KCl concentration. Coordinates of the Hydrogen atoms were generated with CHARMM [44] using standard protonation states for all the titrable residues. Once the system was prepared, 200 ns molecular dynamics (MD) simulations at constant temperature (303.15 K) and pressure (1 bar) were run using NAMD [45], the CHARMM36 force-field [46,47], and Particle Mesh Ewald method [48] to account for the electrostatics of the periodic boundary conditions. A 2 fs time step and the ShakeH [49] algorithm was used. Throughout the 200 ns of MD simulations, all the structures were stable and none of the ligands leave their pocket. A small restrained between the DNA strands was added to the Topo-II simulations to avoid DNA breakage throughout the simulations.

To calculate the docking energy corresponding to each of the ligands, the coordinates of the protein and DNA chains of last frames of the MD simulations were extracted for a subsequent docking stage and carried out using Autodock-Vina [36]. As expected, the most stable pose was equivalent to the coordinates of the ligands in the last frame of the simulation, which validates the docking procedure. To shed light onto the differences among the affinities between the ligands and the proteins, interaction energies were calculated throughout the MD trajectories using NAMD. Parameters for pophyllotoxin, etoposide, podophyllic aldehyde and **4** were assigned using ParamChem [50,51]. The overall procedure was similar to that used in Reference [52].

## 5. Conclusions

In this study, a new conjugate derived from podophyllotoxin, the 7β,9-biscyclolignan **4**, was synthesized and evaluated. Using this natural product as a starting material for preparing the two fragments of the compound, we obtained a novel conjugate that combines the structural requirements for both anti-tubulin polymerization and inhibition of Topo-II activities. The extensive biological analysis carried out with the compounds showed a different behavior for each precursor, and those different behaviors were also observed in the conjugate. The final hybrid **4** achieved the best profile in the viability studies and the highest values in apoptosis induction and in cell cycle arrest at G2/M phase, improving the precursors’ properties. Additionally, the final conjugate had a cytotoxic selectivity in osteosarcoma cells compared to other tumor cell lines. These assays were complemented with molecular docking studies to confirm that the two protein inhibition activities can be involved in the mechanisms of action of the novel conjugate. These results are promising, showing the potential of this 7β,9-biscyclolignan **4**. Further studies that include more structure–activity relationships are currently in progress.

## Figures and Tables

**Table 1:** Docking energies (kcal/mol) calculated for compounds **1**, **2**, **4** and etoposide for tubulin interaction.

Compound	ΔG	Protomers Interaction
Podophyllotoxin (**1**)	−9.5	−496 ± 70
Podophyllic aldehyde (**2**)	−8.5	−449 ± 80
Etoposide	−10.3	−440 ± 80
Hybrid **4**	−12.5	−353 ± 77

**Table 2:** Docking energies (kcal/mol) calculated for compound **1**, **2**, **4** and etoposide for Topo II and DNA interactions.

Compound	ΔG	Interaction Energy Ligand-Protein	Interaction Energy Ligand-DNA
Podophyllotoxin (**1**)	−9.7	−25 ± 11	−41 ± 9
Podophyllic aldehyde (**2**)	−9.4	−27 ± 13	−50 ± 9
Etoposide	−12.4	−37 ± 10	−54 ± 10
Hybrid **4**	−12.8	−62 ± 18	−67 ± 12

## Supplementary Materials

The following are available online, Table S1: Numerical distribution (%) of annexin− and annexin+ cells in the apoptosis assay. Table S2: ^1^H NMR data for compound **3**. Table S3: ^13^C NMR data for compound **3**. Table S4: ^1^H NMR data for compound **4**. Figure S1: ^1^H and ^13^C NMR spectra for compound **3** (CDCl_3_). Figure S2: ^1^H and ^13^C NMR spectra for compound **4** (CDCl_3_).
