# Supplementary material for: A Novel Cytotoxic Conjugate Derived from the Natural Product Podophyllotoxin as a Direct-Target Protein Dual Inhibitor
Source: Molecules. 2020 Sep 17;25(18):4258. doi: 10.3390/molecules25184258 (PMC7571232; doi:10.3390/molecules25184258)
Supplement: Supplementary file 1 [file molecules-25-04258-s001.pdf]

Supplemantary Material

Table S1. Numerical distribution (%) of annexin- and annexin+ cells in the apoptosis assay.

	Cell Line	Annexin -	Annexin +		
			**Total**	**PI-**	**PI+**
**Control**	MG-63	89.58	10.42	33.32	66.68
	HT-29	94.70	5.30	22.55	77.45
	MCF-7	91.86	8.14	17.76	86.24
Podophyllic aldehyde (**2**)	MG-63	36.15	63.85	81.29	18.71
	HT-29	84.17	15.83	84.48	15.52
	MCF-7	83.50	16.50	89.04	10.96
7 -cyclolignan **3**	MG-63	34.98	65.02	86.62	13.38
	HT-29	25.36	74.64	83.63	16.38
	MCF-7	10.24	89.76	21.96	78.04
Hybrid **4**	MG-63	5.32	94.68	82.33	17.77
	HT-29	74.60	25.40	74.60	25.40
	MCF-7	65.23	34.77	14.85	85.15

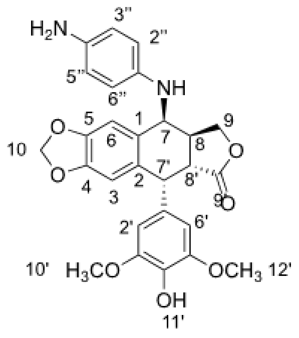

3

Table S2. ^{1}H NMR data for compound **3.**

**H**		**3**
**3**		6.45 *s*
**6**		6.70 *s*
**7**		4.53 *d* (4.8)
**8**		2.85 *m*
**9**		4.29 *m*
		3.09 *m*
**10**		5.90 *s*
**2′6′**		6.26 *s*
**7′**		4.39 *sa*
**8′**		2.79 *m*
**10′ 12′**		3.72 *s*
**linker**	2″, 6″	6.58 *d* (8.0)
	3″, 5″	6.36 *d* (8.0)

Table S3. ^{13}C NMR data for compound **3.**

**C**	**3**

**1**		132.1
**2**		131.8
**3**		110.5
**4**		146.4
**5**		147.4
**6**		108.9
**7**		66.7
**8**		38.1
**9**		67.6
**10**		101.5
**1′**		132.0
**2′6′**		107.8
**3′5′**		148.6
**4′**		134.0
**7′**		43.7
**8′**		40.6
**9′**		175.1
**10′ 12′**		56.5
**linker**	**1″**	147.4
	**2″,6″ 3″,5″**	122.6
	**4″**	148.6

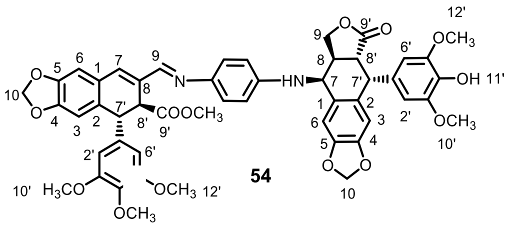

Table S4. ^{1}H NMR data for compound **4.**

**H**	**4** Podophyllic aldehyde rest	linker	4′-desmetyl-epipodophyllotoxin	**H**
**3**	6.61*s*	6.46 *d* (8.8)	6.46 *s*	**3**
**6**	6.73 *s*	7.03 *d* (8.8)	6.70 *s*	**6**
**7**	6.91 *s*		4.53 *d* (5.2)	**7**
**8**			2.85 *m*	**8**
**9**	8.18 *s*		4.30 *m* 3.09 *m*	**9**
**10**	5.91 *s*		5.90 *s*	**10**
**2′6′**	6.28 *s*		6.26 *s*	**2′6′**
**7′**	4.50 *d* (3.2)		4.39 *br s*	**7′**
**8′**	4.36 *d* (3.2)		2.79 *m*	**8′**
**10′ 12′**	3.71 *s*		3.72 *s*	**10′ 12′**
**11′**	3.74 *s*			
**9′-OCH$_3$**	3.60 *s*			

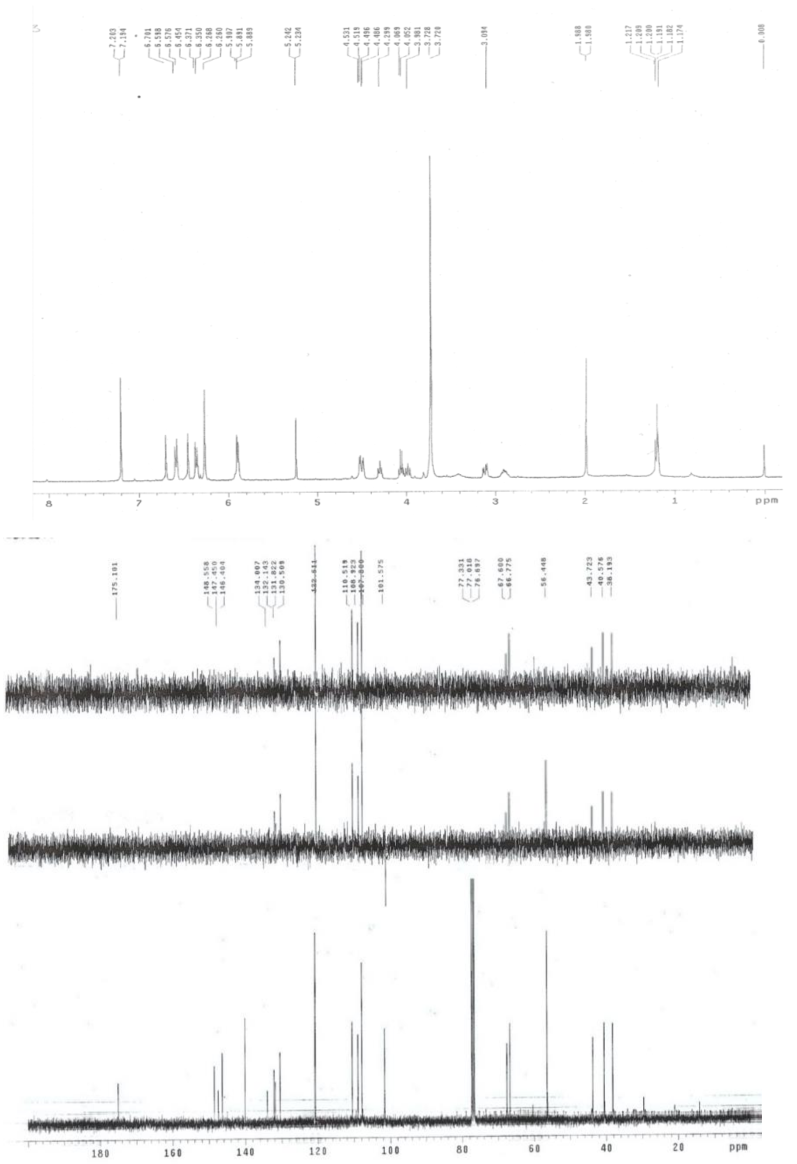

Figure S1. ^{1}H and ^{13}C NMR spectra for compound **3** ($CDCl_3$).

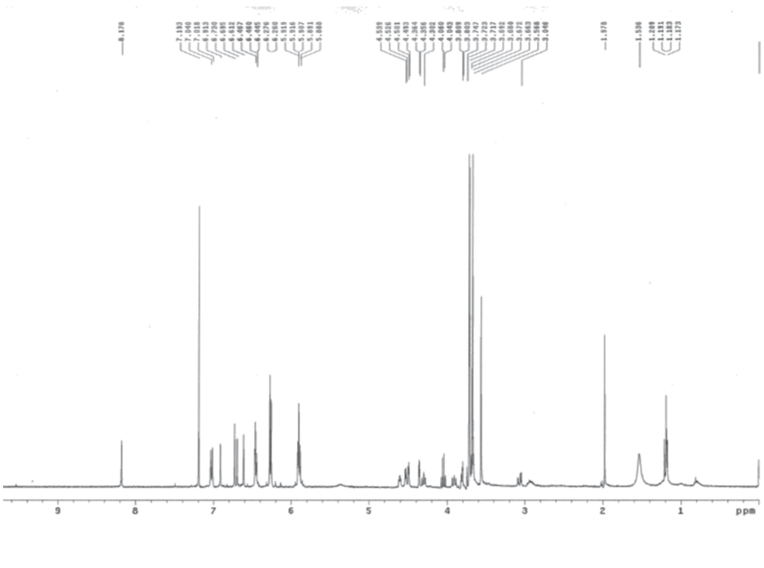

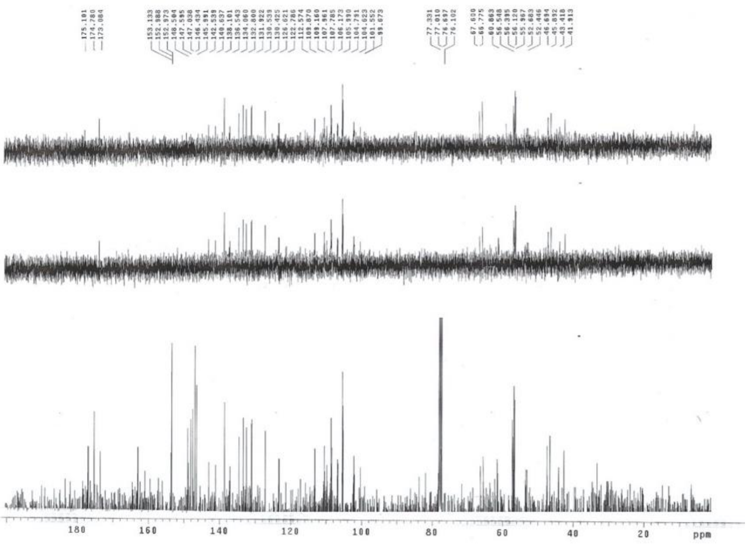

Figure S2. ^{1}H and ^{13}C NMR spectra for compound **4** ($CDCl_3$).
